# Uncovering metabolomic dysregulation in *Lactuca sativa* caused by exposure to nano- and microplastics (NMPs)

**DOI:** 10.3389/ftox.2026.1872426

**Published:** 2026-07-24

**Authors:** María José Gómez-Ramos, Harshit Sahai, Chen Wang, Hui Li, Amadeo R. Fernández-Alba, María Dolores Hernando Guil

**Affiliations:** 1 Department of Chemistry and Physics, Pesticide Residues Research Group, Research Centre for Mediterranean Intensive Agrosystems and Agri-Food, Biotechnology (CIAIMBITAL), University of Almeria, Agrifood Campus of International Excellence, Almeria, Spain; 2 Institute of Environmental Pollution and Health, School of Environmental and Chemical Engineering, Shanghai University, Shanghai, China; 3 Department of Desertification and Geo-ecology, Experimental Station of Arid Zones, Almería, Spain

**Keywords:** edible plants, LC-QTOF-MS/MS, metabolite identification accuracy, metabolomic change, nano- and micro-plastics (NMPs), polyunsaturated fatty acids (PUFAs)

## Abstract

This work investigated the metabolic impact of nano- and microplastics (NMPs) in *Lactuca sativa* using controlled hydroponic exposures combined with non-targeted high-resolution mass spectrometry metabolomics. Polystyrene NMPs of four particle sizes (100, 200, 500, and 1000 nm) were tested across multiple concentrations. Multivariate analysis revealed clear metabolic discrimination between exposed and control plants, with smaller nanoparticles (100–200 nm) inducing the most substantial changes. Among the principal discriminant features, four were selected for in-depth characterization based on their statistical response, all consistently downregulated in NMP-exposed plants. α-Linolenic acid was confirmed at Schymanski Level 1, while three additional C18-class lipid-related features were annotated at Level 4. The four candidate metabolites point to disruption of polyunsaturated fatty acid (PUFA)-related pathways, with the C_18_H_28_O feature showing the strongest size-dependent response. In contrast, α-linolenic acid exhibited a more gradual response across particle sizes, suggesting a different interaction mechanism. Larger particles (500–1000 nm) induced markedly weaker effects, confirming the greater impact of smaller nanoparticles on PUFA downregulation. These findings illustrate the capability of HRMS-based untargeted metabolomics for evaluating NMP-induced metabolic disruption in food crops and its potential implications for plant health and food quality.

## 1 Introduction

Plastic pollution is one of the most pressing environmental issues of the 21st century, impacting diverse ecosystems worldwide. Once primarily seen as a problem for marine and freshwater environments (UNEP, 2021), plastic contamination is now recognized as a significant concern for terrestrial ecosystems, particularly agricultural soils (FAO, 2021). Plastics can enter agricultural systems via multiple pathways, including the application of contaminated sewage sludge or repurposed wastewater from treatment plants (WWTPs), the use of plastic mulch films, and atmospheric deposition (Kaushik et al., 2024; van den Berg et al., 2020; Huang et al., 2020). These plastics degrade into micro- and nanoparticles that can persist in the soil for extended periods (Astner et al., 2019), potentially threatening soil health, plant growth, and food safety.

The presence of microplastics in waters, irrigated crops, and cultivated soil has been recently documented (Sahai et al., 2025; Gao et al., 2024; Jheng-Jie et al., 2023); nevertheless, the relationship between plant morphological alterations and metabolomic responses remains insufficiently explored, highlighting significant gaps that require further investigation. The potential for plastic particles to be absorbed by plants and subsequently accumulate in edible crops has raised significant concerns regarding their entry into the food chain. To date, only a limited number of studies have demonstrated that certain types of nanoparticles can be absorbed through plant roots (Sahai et al., 2024; Kim et al., 2022; Liu et al., 2022; Dong et al., 2021; Gong et al., 2021; Li et al., 2021; Zhou et al., 2021; Li et al., 2020; Sun et al., 2020; Giorgetti et al., 2020; Zhang et al., 2019; Jiang et al., 2019), via foliar application (Lian et al., 2021; Sun et al., 2021), or translocated to aerial tissues such as leaves (Sahai et al., 2024). The behavior of nano- and microplastics (NMPs) within plant systems remains poorly understood, particularly in terms of their uptake, translocation, and physiological impacts.

Beyond understanding the uptake of NMPs by plants, it is essential to explore their metabolic impacts. Plant metabolomics can link stress effects caused by environmental contaminants, providing insights into alterations in primary and secondary metabolites (Cabrera-Peralta and Peña-Alvarez, 2024). Previous studies have shown that environmental factors such as temperature and light, and agricultural practices can influence the content of bioactive compounds such as flavonoids, phenolic acids, carotenoids, vitamins, tocopherols, and sesquiterpene lactones related to lettuce and other cultivate plants quality (Yang et al., 2022; Martínez Bueno et al., 2018). Long-term exposure to plastic particles, even at low concentrations, could interfere with key metabolic processes, affecting plant growth, nutrient absorption, and stress resilience. Recent research predominantly employs GC-MS and LC-MS coupled with established spectral libraries and specialized data analysis platforms such as MetaboAnalyst to perform untargeted metabolomics analyses in various crops, including rice, wheat, cucumber, cabbage, radish, stevia, dandelion, spinach or maize (Lian et al., 2020; Iqbal et al., 2024; Huang et al., 2023; Guo et al., 2023; Fan et al., 2022).

As in the case of GC-MS-based studies on wheat exposed to polystyrene nanoparticles (PSNPs) have identified changes in 59 metabolites affecting energy and amino acid metabolism pathways, such as the TCA cycle and galactose metabolism (Lian et al., 2020). Similarly, rice grown under combined stress of invasive species and polyethylene MPs, revealed over 200 metabolites altered, including amino acids, lipids, and carbohydrates, indicative of an enhanced antioxidant response (Iqbal et al., 2024). Cucumber leaves exposed to PSNPs demonstrated significant shifts in carbohydrate and amino acid metabolites, identified by GC-MS after derivatization (Huang et al., 2023). Other studies employing LC-MS/MS, have reported metabolic disruptions in amino acid biosynthesis and phosphorylation processes in barley and aquatic plants exposed to MPs and nanoparticles (Guo et al., 2023; Fan et al., 2022).

While these studies have advanced the understanding of how MPs impact plant metabolism, they largely rely on matching spectral data to known libraries, an approach that enables broad profiling but is constrained by the availability and coverage of reference spectra. This gap is significant, as metabolomic profiling can detect subtle metabolic shifts indicative of stress responses or disruptions not evident through traditional physiological assessments. A metabolomic approach is particularly valuable for studying NMPs exposure, as these particles can interact directly with cellular components, potentially disrupting enzyme activity, metabolic cycles, and nutrient assimilation. The resulting alterations in metabolic pathways may affect vital compounds such as amino acids, carbohydrates, lipids, and antioxidants, which are critical for plant health, resilience, nutritional quality, and food safety.

This study investigates the uptake and translocation of nano- and microplastics (NMPs) in *Lactuca sativa* and assesses the plant’s metabolic response. Plants were exposed to NMPs ranging from 100 to 1000 nm to determine whether particle size affects uptake and translocation within plant tissues, and the metabolic impact was examined by untargeted profiling. Using high-resolution LC-QTOF-MS/MS, this non-targeted analysis reveals shifts in metabolites associated with stress responses, providing both critical insights into the metabolic dysregulation caused by plastic NMPs and a proof-of-concept for resolving the complex interactions between NMPs and plants. To our knowledge, the simultaneous evaluation of four polystyrene NMP particle sizes (100, 200, 500 and 1000 nm) across four concentration levels by untargeted LC-QTOF-MS/MS metabolomics in hydroponically grown *L. sativa* has not been previously reported; earlier plant NMP metabolomics studies have typically examined a single particle size or polymer, applied targeted or widely-targeted approaches, or focused on soil and root systems rather than the leaf metabolome. By combining controlled hydroponic cultivation with metabolomic analysis, and by examining both the morphological and metabolomic impacts, this study contributes foundational knowledge on the risks posed by plastic NMPs contamination in agricultural settings.

## 2 Materials and methods

### 2.1 Reagents and materials

Polystyrene NMPs stock solutions were purchased from Applied Microspheres (Applied Microspheres BV, Netherlands), supplied as an aqueous suspension (1% solids). According to the supplier information, the solutions also contained <0.1% undisclosed surfactants and <0.05% preservatives. The stabilizing surfactant present in the polystyrene suspensions was subsequently identified as polyethylene glycol ethoxylates (PEG) by mass-spectrometric analysis. Based on the supplier specification (<0.1% of solids), the surfactant introduced is at most 50 μg/L at the highest exposure (50 mg/L NMP) and at most 0.1 μg/L at 100 μg/L, with the preservative load approximately half these values. The particles were neutrally charged and devoid of any surface functionalization. The nominal diameters were 0.100 μm (100 nm; mean 0.098 μm), 0.200 μm (200 nm; mean 0.198 μm), 0.500 μm (500 nm; mean 0.498 μm) and 1 μm (1000 nm; mean 0.998 μm), with CV < 5% in all cases. Hoagland plant nutrient mix (powder) was purchased from Sigma-Aldrich (United States) and diluted per supplier instructions to prepare the hydroponic nutrient solution. The plants were obtained as commercial *L. sativa* L. seedlings at the four-true-leaf stage from a local agricultural supplier.

For the extraction and analysis of metabolites, an automatic axial extractor (AGYTAX®) from Cirta Lab S.L (Madrid, Spain) and a centrifuge from Ortoalresa (Madrid, Spain) were used. LC-MS (liquid chromatography-mass spectrometry) grade ultra-pure water was obtained from Fisher Scientific (Fair Lawn, New Jersey, USA), and LC-MS grade methanol from Fluka Analytical (Steinheim, Germany). Formic acid (99% purity) was acquired from Fluka (Buchs, Switzerland) and ammonium formate from Sigma-Aldrich (Steinheim, Germany). The electrospray ionization (ESI) positive calibration solution X500 was provided by AB SCIEX™ (Framingham, MA, USA).

### 2.2 Plant exposure experiments

*Lactuca sativa* was chosen as the model plant for its short life cycle and suitability to hydroponic culture, which offers precise control over nutrients and exposure and minimizes external variability. Experiments tested whether uptake is size-dependent (100–1,000 nm) and whether metabolism is altered. All steps, including stock-solution preparation, plant growth, and sample storage, transport and processing, used glass apparatus to eliminate ambient and cross-contamination. For each combination of size and concentration, groups of 7 plants were exposed to 30 mL of Hoagland nutrient solution containing 10 μg/L, 100 μg/L, 300 μg/L or 50 mg/L polystyrene NMPs (μg/L equivalent to ppb and mg/L to ppm hereafter). Reported soil microplastic concentrations span ∼1 to 80,000 particles kg^-1^, highest in sludge-amended and plastic-mulched soils (Sahai et al., 2025); as these are mostly particle counts for microplastics whereas our exposures are mass-based and in the nanoplastic range, direct equivalence was not possible and reliable nanoplastic field values remain scarce. Accordingly, our lower exposures (10–300 μg L^-1^) approximate the upper range of plausible environmental exposure in intensively managed, irrigated systems, while the highest dose (50 mg L^-1^) was a deliberately aggressive, accelerated-exposure scenario rather than a typical field level.

A control group of 7 plants was grown in identical Hoagland solution without NMPs under the same incubator, glassware, photoperiod and handling as every treatment, serving as the unexposed reference for all comparisons. Exposure lasted 20 days at 24 °C, 60% relative humidity and a 12 h photoperiod, with the nutrient solution refilled every second day to maintain a constant 30 mL volume. Plant positions were re-randomized at every renewal, distributing each treatment across all incubator positions and averaging out spatial gradients in temperature, light and air circulation rather than confounding them with treatment. The experimental unit was the individual plant for morphological analyses and the pooled biological replicate for metabolomics. The sample size (n = 7 independent biological replicates per treatment) was based on biomass requirements for extraction and growth-chamber capacity and is consistent with comparable untargeted metabolomics studies of plant stress responses.

### 2.3 Confocal laser-scanning microscopy analysis of plant tissues

The detailed methodology of analysis is published elsewhere (Sahai et al., 2024). Briefly, plant samples were divided into roots and leaves post-exposure to fluorescent polystyrene nanoplastics to validate root uptake and subsequent translocation to aerial tissues. The separated components were rinsed by gently agitating them under a narrow stream of running ultrapure filtered water thrice (each cycle up to 1 min) to remove superficial particles that were not absorbed or were loosely attached.

The sectioned tissues were examined by confocal laser scanning microscopy (Leica TCS SP8; LAS X software), with fluorescence recorded at an excitation/emission of 530/580 nm. Control (unexposed) plants were imaged under identical acquisition settings, and their red-channel signal was used to define and subtract the background-autofluorescence threshold, so that reported signals represent particle fluorescence above tissue autofluorescence. Internalization rather than surface attachment was established by confocal optical sectioning, with Z-stacks acquired through the tissue localizing the fluorescence signal within root and leaf tissue rather than at the surface. The confocal analysis provided qualitative confirmation of NMP uptake and root-to-leaf translocation, complementing the metabolomic analysis that is the central focus of this work; quantitative imaging would require a larger set of replicate images than were acquired here and was beyond the present scope. Full CLSM acquisition settings, image processing details using Fiji-ImageJ and instrument QA/QC are given in Supplementary Table S1.

### 2.4 Extraction procedure

Each extract consisted of a pool of up to 7 plants from the same treatment. Natural plant losses during drying and processing resulted in some pools being prepared from 4 to 7 plants; since each pool constitutes a single biological observation regardless of contributing plant number, and all pools were prepared under identical conditions, this variation does not introduce systematic bias. Statistical analyses used Welch t-tests, which do not assume equal group sizes or variances. Immediately after harvest, root and leaf tissues were frozen and stored at −80 °C and subsequently lyophilized. Crushed, homogenized, and sieved lettuce plant material (0.5 g) was weighed into 15 mL PTFE falcon tubes. To each tube, 15 µL of a 10 mg/L mixture of three isotopically labeled procedural internal standards were added. Following this, 2.5 mL of ultrapure water was added to each tube, and the samples were left to settle for 5 min. The extraction process began by adding 2.5 mL of methanol to each tube. The samples were shaken for 30 min using an automatic axial agitator. Subsequently, the samples were centrifuged at 5000 rpm for 30 min, and the supernatant was transferred to a 2 mL amber glass vial. Before injection, a 400 µL aliquot of the extract was transferred to a 0.2 µm PTFE filter vial where it was simultaneously filtered. Then, 10 μL of the internal injection standard (0.1 mg/L) was directly added to the sample.

### 2.5 Analysis by LC-QTOF-MS/MS

An ExionLC™ (SCIEX, Massachusetts, USA) UHPLC instrument was coupled with an X500R tandem quadrupole time-of-flight (QTOF) high-resolution mass spectrometry (HRMS) instrument equipped with a Turbo V™ Source with a TwinSprayer probe (AB SCIEX™) and using an electrospray ionization source (ESI).

Liquid chromatographic separation used an Agilent Zorbax Eclipse Plus C8 column (100 × 2.1 mm, 1.8 µm) with a C18 guard column, and water (A) and methanol (B) mobile phases, each 98:2 (v/v) with the minor solvent and containing 5 mM ammonium formate and 0.1% formic acid. The flow rate, column temperature, injection volume and the complete elution gradient are given in Supplementary Table S2. The ESI interface was operated in positive ionization mode, with the TOF system providing a resolving power of 32,000 fwhm at *m/z* 200. HRMS data were acquired in full-scan information-dependent acquisition (IDA) mode, combining a TOF-MS survey scan (*m/z* 80–950) with dependent MS/MS scans (*m/z* 50–950). The ESI source parameters, IDA selection criteria, declustering potential, collision energy and accumulation time are also listed in Supplementary Table S2. Automatic external mass calibration was injected with the batch every five samples, maintaining mass accuracy within 2 ppm.

The formation of [M+CH_3_OH+H]^+^ adducts under the ESI conditions employed was systematically confirmed for the four candidate compounds reported in this study. Adduct identity was confirmed by three independent criteria: (i) automatic [M+CH_3_OH+H]^+^ ion type assignment by SCIEX OS; (ii) co-elution of the [M+H]^+^ precursor and [M+CH_3_OH+H]^+^ adduct at identical retention time in XIC overlays; and (iii) the diagnostic neutral loss of 32.026 Da (CH_3_OH) in MS/MS spectra. Full spectral evidence for all four compounds is provided in Supplementary FIgures S1, S2. The [M+CH_3_OH+H]^+^ adduct ion was used as the primary ion for statistical comparison across groups due to its superior chromatographic integration consistency in biological matrix samples.

### 2.6 Data analysis

#### 2.6.1 Data acquisition and processing

Data were acquired and processed in Explorer and Analytics software (v3.0.0.3, AB Sciex). Compound confirmation used both precursor and fragment ions, with elemental compositions assigned by the formula-finder tool (C, H, N, O, S) and supported by isotope-pattern distribution, ring-and-double-bond equivalents and MS/MS interpretation. The peak-detection, mass-accuracy and formula-assignment thresholds are given in Supplementary Table S3.

#### 2.6.2 Non-target screening strategy. Feature detection and alignment

For non-target screening, a case-control data processing approach was employed. Control consisted of lettuces not exposed to NMPs, while case samples were those exposed to NMPs. Both sets of samples were analyzed simultaneously to minimize variability. Feature finding and data alignment were performed in MarkerView software (v1.3.1, SCIEX), generating an HRMS peak list for each sample; the peak-detection and alignment parameters are listed in Supplementary Table S3. Furthermore, the pre-processing steps and statistical analysis protocol are provided in detail in Supplementary Text S1.

#### 2.6.3 In silico fragmentation

To assist in compound identification, Mass Frontier Spectral Interpretation software 8.0 (Thermo Scientific™) was used to generate *in silico* fragmentation patterns and structures. These computationally generated spectra were compared to the experimental IDA MS/MS spectra for structural confirmation.

### 2.7 Quality control

Procedural blanks and instrumental blanks were analyzed alongside each batch injection to assess potential cross-contamination, analyte carryover and background response. Internal standards were employed to ensure the quality of the measurements. A solution of dimethoate-D6 was used as the injection standard, while a mix of carbendazim-D3, dichlorvos-D6, and malathion-D10 were used as procedural internal standards. The internal standards used in this study were selected for overall analytical quality control rather than for class-specific matrix effect compensation. Isotopically labelled lipid-class standards were not available for this study; this represents a limitation for absolute quantification of PUFA-related features, although relative comparisons between groups processed under identical conditions are not substantially affected.

Quality control (QC) samples were prepared by combining identical aliquots of all sample extracts. These QC samples were analyzed alongside the real samples, with injections performed at three points during the analysis sequence: at the beginning, middle, and end.

## 3 Results and discussion

### 3.1 Changes to plant morphological traits in response to polystyrene NMPs exposure

**FIGURE 1:**
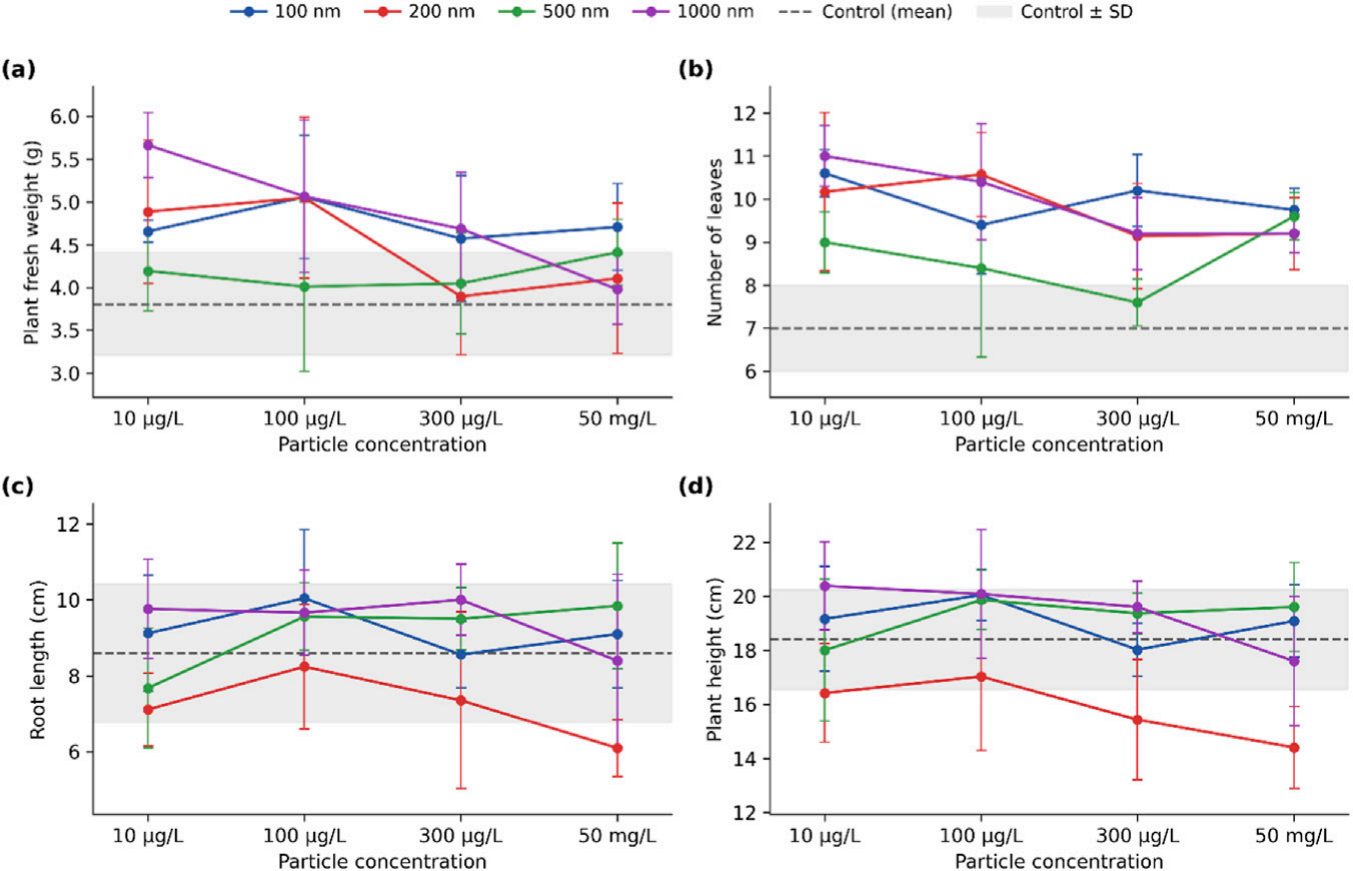
Changes in morphological traits of Lactuca sativa after 20-day hydroponic exposure to polystyrene NMPs. Plant fresh weight **(a)**, number of leaves **(b)**, root length **(c)** and plant height **(d)**, shown as mean ± SD for each particle size (100, 200, 500 and 1,000 nm) across the four exposure concentrations (10 μg/L, 100 μg/L, 300 μg/L and 50 mg/L).

At the end of the 20-day exposure, fresh weight, height, root length and leaf number were recorded for every plant and compared against the unexposed control (Figure 1). Detailed treatment-wise mean and SD values are provided in Supplementary Table S4 for our readers. The data were analyzed in two complementary ways: a two-way ANOVA partitioned the variance of the sixteen size × concentration treatments between particle diameter and concentration (using Type III sums of squares, appropriate for the unequal group sizes, n = 4–7), and a one-way ANOVA across all seventeen groups, followed by Tukey’s HSD, provided the treatment-versus-control and pairwise contrasts. Particle diameter was the dominant source of variation for every trait, explaining 41% of the variance in height (F (3, 68) = 19.75, p < 0.0001), 29% in root length (F (3, 68) = 11.38, p < 0.0001), 17% in leaf number (F (3, 68) = 6.94, p = 0.0004) and 10% in fresh weight (F (3, 68) = 3.87, p = 0.013). Concentration was weaker and less consistent: it was significant only for fresh weight (11% of variance, p = 0.012) and leaf number (11%, p = 0.008), and not for height (p = 0.052) or root length (p = 0.11). A size × concentration interaction reached significance only for leaf number (p = 0.043).

Compared to the control samples, the response was one of growth maintenance and, in two traits, stimulation rather than inhibition. Control plants carried the fewest leaves of any group (7.0 ± 1.0), and eight of the sixteen treatments produced significantly more, the largest gains at the lowest concentrations of the smallest and largest particles (1000 nm at 10 μg/L, 11.0 leaves, p < 0.0001; 200 nm at 100 μg/L, 10.6, p < 0.0001; 100 nm at 10 μg/L, 10.6, p = 0.0001). Fresh weight moved the same way: every treatment´s mean exceeded the control (3.81 ± 0.60 g), and the heaviest plants, 1000 nm at 10 μg/L, 5.66 g were significantly larger, the only treatment to reach significance against the control (p = 0.006).

The cost of exposure appeared in height and root length, and it was confined to the 200 nm size class. After multiplicity correction, no treatment was significantly shorter, or shorter-rooted, than the control. The significant pairwise differences came entirely from the 200 nm plants and increased with dose. At 50 mg/L the 200 nm plants were the shortest in the experiment (14.4 cm, against 18.4 cm in the control) and shorter than the larger-particle treatments by up to 6.0 cm (versus ,1000 nm at 10 μg/L, p = 0.0003); the same plants had the shortest roots (6.1 cm, against 8.6 cm), significantly shorter than several 100, 500 and 1000 nm treatments by 3.5–3.9 cm (p < 0.05). The 100, 500 and 1000 nm particles matched or slightly exceeded the control for both traits. The size response was therefore non-monotonic: benign at 100 nm, suppressive at 200 nm, neutral-to-positive at 500 and 1000 nm rather than a smaller-is-worse gradient.

Read together, the four traits separate into two coordinated groups. Height and root length behaved as architectural traits, governed almost entirely by particle size, insensitive to concentration, and sharing a single minimum at 200 nm where plants were both the shortest and the shortest-rooted. That shoot and root length fell and rose together (short roots with short shoots at 200 nm, longer roots with taller shoots at 500–1,000 nm) points to a root-limited control of shoot elongation rather than independent effects on each organ. Leaf number and fresh weight behaved as productivity traits: relative to the unexposed control they were raised rather than depressed, every treatment carrying more leaves and equal-or-greater biomass, and they were the only traits for which exposure concentration carried a measurable effect. The whole-plant response is therefore not one of toxicity but of a size-tuned reallocation, with elongation held back specifically by 200 nm particles while leaf production and biomass were broadly stimulated.

The dependence on concentration was non-monotonic but weak and inconsistent. A size-dependent dose–response shape was significant only for leaf number (p = 0.043), where the 200–1000 nm particles reached a local minimum at 300 μg L^-1^ and partly recovered at 50 mg L^-1^, echoing the 300 μg L^-1^ inflection seen in the metabolome in subsequent sections; the other three traits showed no size-dependent shape. Two non-exclusive mechanisms could produce this behavior: at higher mass loadings the particles aggregate into larger, less colloidally stable clusters that lower the bioavailable particle number and effective root uptake, so the internal dose need not scale with the nominal concentration; and a sub-injurious particle load can trigger compensatory growth, the signature of a hormetic response in plant–nanoparticle systems (Bell et al., 2013). Low concentrations were not inert: the most leaves (11.0) and the greatest biomass (5.66 g) of the whole experiment occurred at the lowest concentration of 1000 nm particles, with biomass under 1000 nm falling monotonically as concentration rose. We therefore treat these dose–response shapes as exploratory, pending particle-size characterization across the concentration range in the exposure medium and a denser dose series. What is unambiguous is that particle size, not concentration, sets the morphological response, and that the metabolic perturbation reported below is the more sensitive readout, evident even at sizes that left the plant’s external form intact or enhanced.

These effects can be attributed to the bioaccumulation of the exposed NMPs in the plant tissues, as confirmed by complementary fluorescence-based confocal laser scanning microscopy (CLSM). The analysis verified that NMPs ranging from 100 nm up to 1,000 nm were accumulated within plant tissues and also effectively translocated from roots to leaves. Representative CLSM images including controls and those highlighting the accumulation of NMPs in *Lactuca sativa* tissues are provided in the Supplementary Material (Supplementary Figures S3-S6).

### 3.2 Metabolomic profiling of Lactuca sativa plants exposed to NMPs and metabolite identification

To assess the metabolic changes in *L. sativa* plants induced by NMPs exposure, an untargeted analysis followed by unsupervised and supervised chemometric analysis was conducted. Following pre-processing (total area sum normalization, isotope removal, blank subtraction, and 80% prevalence filtering), the initial feature list was reduced to 1,630 high-confidence features across 14 control and 25 NMP-exposed samples. Analytical reproducibility, assessed using pooled QC samples, was excellent: the mean CV% across retained features was 14.3% (median 11.4%), with 91.8% of features showing CV% < 30%. CV% values were used as a quality metric; all 1,630 features were retained for statistical analysis. QC clustering in the unsupervised PCA scores plot (Supplementary Figure S7A) further confirmed analytical stability throughout the injection sequence.

**FIGURE 2:**
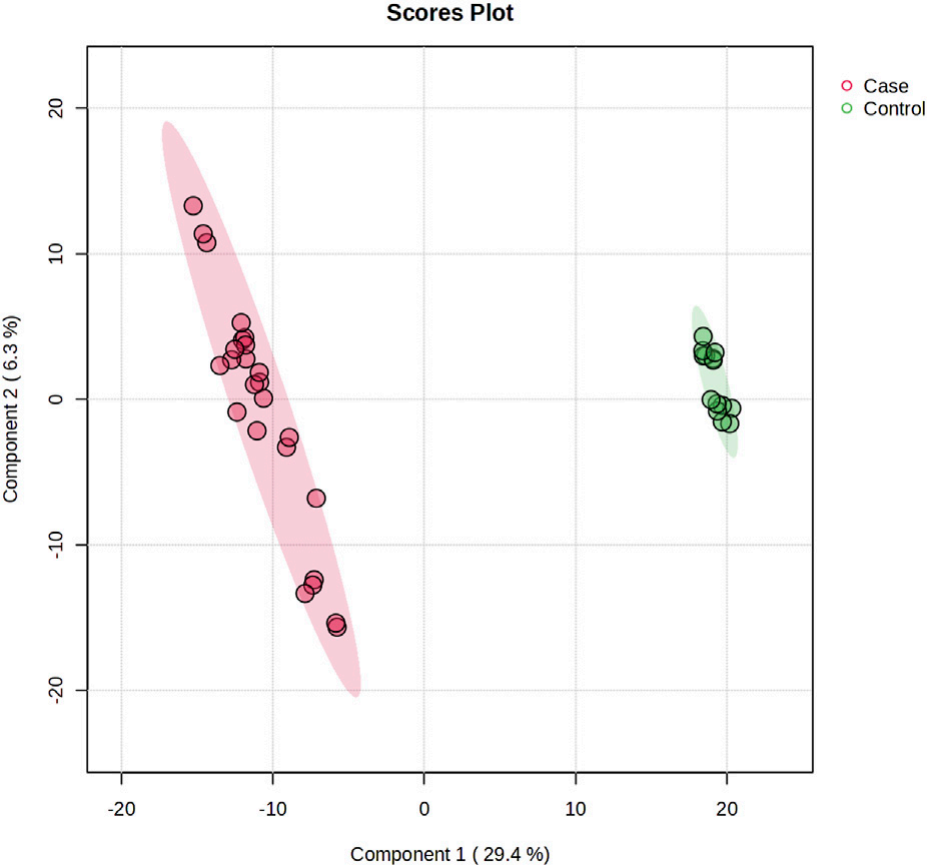
PLS-DA score plot of *Lactuca sativa* plants exposed to polystyrene NMPs and control plants not exposed.

Unsupervised Principal Component Analysis (PCA) was carried out to explore and visualize overall metabolic differences between the plants exposed to polystyrene NMPs and the non-exposed control plants (Supplementary Figures S7A, S7B). To further investigate these effects, Partial Least Squares Discriminant Analysis (PLS-DA), Welch t-test, and visualization tools such as volcano plots and profile plots were employed to identify specific metabolites that significantly differed between the two groups. The PLS-DA model yielded R^2^ = 0.998 and Q^2^ = 0.991 with 2 components (R^2^−Q^2^ < 0.01), indicating robust class discrimination with no evidence of substantial overfitting (Supplementary Figure S8B). The Welch t-test with Benjamini-Hochberg FDR correction identified 807 features significant at q < 0.05. The PLS-DA score plot in Figure 2 shows the distinct clustering of NMP-exposed and control samples.

**FIGURE 3:**
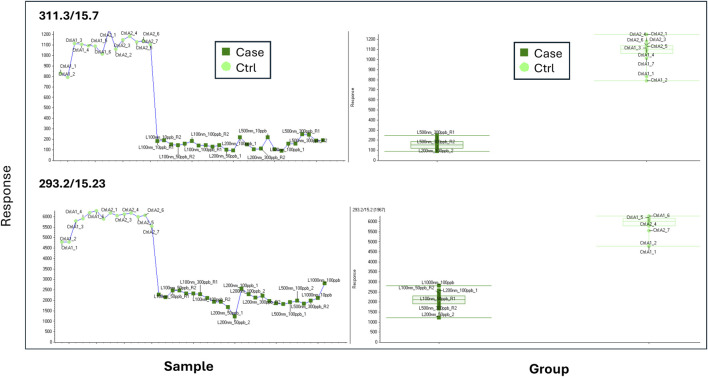
Profile and box-and-whisker plots for candidate ions *m/z* 311.2575 at retention time (Rt) 15.71 min and *m/z* 293.2469 at Rt 15.23 min.

A volcano plot was used to identify statistically significant features, characterized by low p-values and large fold changes, appearing at the extremes of the plot. Due to the large number of significant features, a subset of approximately 30 candidate ions was selected based on their higher signal intensity (peak area) and lower q-values, representing those most strongly associated with NMP exposure. Profile and Box and Whisker plots were employed to examine the distribution of the selected masses across all samples (Figure 3), confirming that the candidate ions were present at significantly higher levels in control plants than in NMP-exposed plants, indicating their downregulation in response to NMP exposure.

To identify the discriminating components, the selected masses were linked back to the raw mass spectra. The candidate list was further refined by assigning adducts, eliminating signals likely resulting from in-source fragmentation, evaluating MS and MS/MS spectral quality, and assigning probable molecular formulas using chemical databases. This process resulted in a final list of four candidate compounds, either identified or tentatively annotated, which are presented in Table 1. The table includes the proposed identification, retention time (Rt), molecular formula, mass error (ppm), MS and MS/MS scores, ring and double bond equivalents (RDB), main adducts (with precursor [M+H]^+^ ions highlighted in bold and the most abundant ion in MS1 marked with an asterisk), accurate mass measurements of molecular and fragment ions (with relative abundances, R.A.), elemental composition assignments, fold change, FDR q-value, LIPID MAPS LMSD candidate counts, and identification confidence level. At least six fragment ions were considered for each compound, with mass errors below 10 ppm and in most cases below 5 ppm.

**TABLE 1:** Metabolites identified or tentatively identified as discriminant by LC-QTOF-MS/MS in *Lactuca sativa* exposed to polystyrene NMPs.

Rt (min)	Molecular formula	Score (%)/ MS error (ppm)/ RDB	Precursor ion/Additional adducts	Fragment ions; (R.A., %); *Elemental composition*	Fold change	FDR q-value	LIPID MAPS	ID level^+^
15.71	C_18_H_30_O_2_	83.1/ 1.8/ 3	279.23186 [M+H]^+^ 296.2584 [M+NH4]^+^ 301.2141 [M+Na]+ 311.2575 [M+CH_3_OH+H]^+^ *	67.0542; (100); *C*_*5*_ *H*_*7*_^*+*^ 81.0704; (72); *C*_*6*_ *H*_*9*_^*+*^ 55.0547; (67); *C*_*4*_ *H*_*7*_^*+*^ 95.0855; (50); *C*_*7*_ *H*_*11*_^*+*^ 279.2318; (50); *C*_*18*_ *H*_*31*_ *O*_*2*_^*+*^ 125.0971; (39); *C*_*8*_ *H*_*13*_ *O* ^*+*^ 261.2230; (32); *C*_*18*_ *H*_*29*_ *O* ^*+*^ 161.0957; (32); *C*_*12*_ *H*_*17*_^*+*^	7.16	7.05 × 10^−25^	α-linolenic acid Level 1	1
15.12	C_18_H_28_O_2_	71/ 2.9/ 4	**277.21621 [M+H]** ^**+**^ 309.2415 [M+CH_3_OH+H]^+^ ^*^	81.0699; (100); *C*_*6*_ *H*_*9*_^*+*^ 151.1117; (9V4); *C*_*10*_ *H*_*15*_ *O* ^*+*^ 67.0542; (80); *C*_*5*_ *H*_*7*_^*+*^ 95.0855; (75); *C*_*7*_ *H*_*11*_^*+*^ 277.2162; (61); *C*_*18*_ *H*_*29*_ *O*_*2*_^*+*^ 55.0542; (40); *C*_*4*_ *H*_*7*_^*+*^ 133.1012; (32); *C*_*10*_ *H*_*13*_^*+*^ 107.0855; (29); *C*_*8*_ *H*_*11*_^*+*^ 165.1274; (23); *C*_*11*_ *H*_*17*_ *O* ^*+*^ 231.2107; (14); *C*_*17*_ *H*_*27*_^*+*^	3.82	6.60 × 10^−19^	70 Candidates (59-unsaturated fatty Acids)	4
14.65	C_18_H_26_O_2_	73/ 1.2/ 6	**275.20056 [M+H]** ^**+**^ 307.2264 [M+CH_3_OH+H]^+^ ^*^	79.0540; (100); *C*_*6*_ *H*_*7*_^*+*^ 55.0542; (79); *C*_*4*_ *H*_*7*_^*+*^ 149.0961; (78); *C*_*10*_ *H*_*13*_ *O* ^*+*^ 93.0702; (45); *C*_*7*_ *H*_*9*_^*+*^ 107.0855; (44); *C*_*8*_ *H*_*11*_^*+*^ 67.0549; (34); *C*_*5*_ *H*_*7*_^*+*^ 125.0963; (34); *C*_*8*_ *H*_*13*_ *O* ^*+*^ 275.2006; (29); *C*_*18*_ *H*_*27*_ *O*_*2*_^*+*^ 229.1951; (6); *C*_*17*_ *H*_*25*_^*+*^ 257.1905; (6); *C*_*18*_ *H*_*25*_ *O* ^*+*^	4.3	2.61 × 10^−20^	22 Candidates (14-unsaturated fatty Acids)	4
15.23	C_18_H_28_O	72/ 2.8/ 4	**261.22129 [M+H]** ^**+**^ 293.2469 [M+CH3OH+H]^+^ *	81.0699; (100); *C*_*6*_ *H*_*9*_^*+*^ 67.0542; (99.9); *C*_*5*_ *H*_*7*_^*+*^ 95.0855; (64); *C*_*7*_ *H*_*11*_^*+*^ 109.1012; (16); *C*_*8*_ *H*_*13*_^*+*^ 121.1012; (8); *C*_*9*_ *H*_*13*_^*+*^ 135.1168; (6); *C*_*10*_ *H*_*15*_^*+*^	2.9	3.80 × 10^−23^	0 Fatty acid candidates**	4

* Most abundant ion detected in MS1 spectrum, used for statistical comparison across groups. ** No fatty acid candidates in LIPID, MAPS LMSD, for C_18_H_28_O.

^+^ Schymanski´s levels: Level 1, confirmed by reference standard; Level 4, unequivocal molecular formula only. Fold change: Control/Case ratio (values >1 indicate downregulation in NMP-exposed plants). FDR: Benjamini-Hochberg correction applied to 1,630 features (Welch t-test, MetaboAnalyst 6.0). Rt- Retention time; R.A.- relative abundance; RDB: rings and double bonds equivalent.

Compound identification confidence was assessed using Schymanski classification framework (Schymanski et al., 2014). α-Linolenic acid (C_18_H_30_O_2_, RT = 15.71 min) was confirmed at Schymanski Level 1 by exact mass (MS error = < 2 ppm), molecular formula isotope profile score (>80), and MS/MS library Purity >95% against the authentic reference standard (Supplementary Figure S1). In the analytical standard, the [M+H]^+^ ion at m/z 279.232 was the most abundant species; however, in *Lactuca sativa* extracts the [M+CH_3_OH+H]^+^ adduct at m/z 311.258 predominated, consistent with matrix-enhanced adduct stabilization under ESI conditions; its identity as methanol adduct was confirmed by neutral loss of 32.026 Da in MS/MS and by its co-elution with the [M+H]^+^ precursor at identical retention time (Supplementary Figure S1B).

The three remaining discriminant features were annotated at Schymanski Level 4 (unequivocal molecular formula only). A systematic search of LIPID MAPS LMSD revealed 70 candidate structures for C_18_H_28_O_2_ (59 unsaturated fatty acids, RT = 15.12 min), 22 candidates for C_18_H_26_O_2_ (14 unsaturated fatty acids, RT = 14.65 min). Given the large number of structurally plausible candidates for each formula, a comprehensive evaluation of all candidates against the observed MS/MS fragmentation patterns was not feasible; specific structural assignment was therefore not attempted. For C_18_H_28_O (RT = 15.23 min), no fatty acid candidates were found in LIPID MAPS LMSD; two isoprenoid entries were retrieved but excluded based on incompatibility with the observed MS/MS fragmentation pattern, which is consistent with a linear unsaturated C18 chain. The molecular formula (single oxygen atom, RDB = 4) is consistent with a C18 unsaturated aldehyde or ketone but given the absence of database candidates and uncertainty regarding its metabolic origin, this feature is conservatively annotated as an uncharacterized C18 oxygenated metabolite whose biological significance warrants further investigation. For all three compounds [M+CH_3_OH+H]^+^ adduct confirmation is provided in Supplementary Figure S2.

**FIGURE 4:**
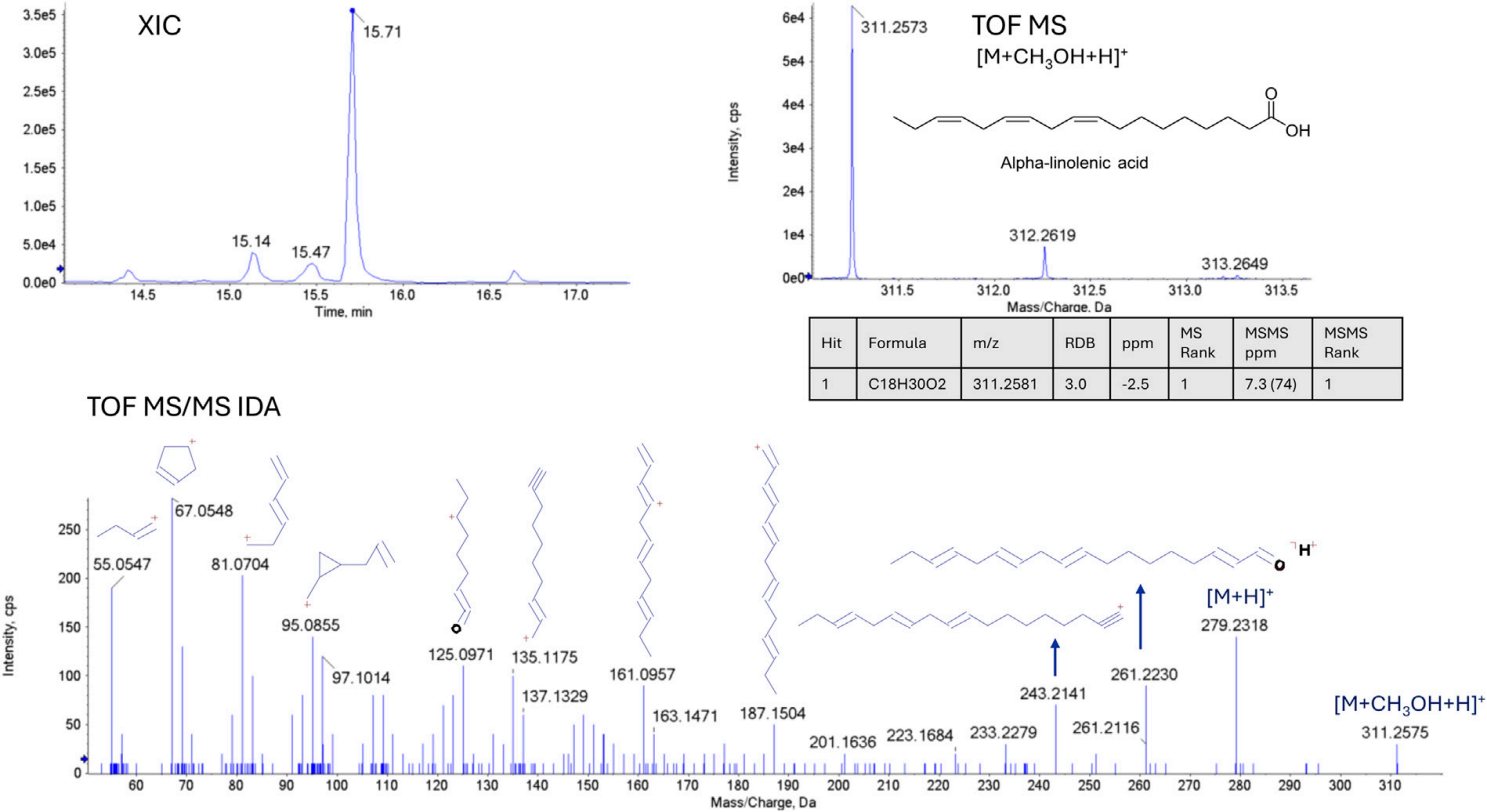
Identification of α-linolenic acid in *Lactuca sativa* extracts by LC-QTOF-MS/MS analysis. The figure includes the extracted ion chromatogram (XIC) at m/z 311.258, the TOF-MS spectra showing the [M+CH_3_OH+H]^+^ adduct as the most abundant ion, and the TOF-MS/MS spectra with the proposed structures for the main observed fragment ions, showing the characteristic C18 polyunsaturated fatty acid fragmentation series. Identity confirmed at Schymanski Level 1.

The identification workflow combined TOF-MS and TOF-MS/MS data interpretation with computational tools such as MetFrag and Mass Frontier™ Spectral Interpretation Software, along with chemical databases including PubChem, MassBank, ChemSpider, and LIPID MAPS LMSD, supplemented by bibliographic searches. Candidate compound characterization was conducted in two main steps: (i) determination of elemental composition based on accurate mass measurements, incorporating isotope pattern analysis, mass defect, and ring and double bond equivalents to narrow down possible candidates; and (ii) structure elucidation using computational tools and public spectral libraries to match MS/MS fragmentation data with proposed molecular formulas. Figure 4 shows the identification details of α-linolenic acid (Level 1), including the extracted ion chromatograms (XIC), full-scan mass spectrum (TOF-MS), fragment ion spectrum (TOF-MS/MS), and isotopic profile. In-silico fragmentation, supported by spectral interpretation software and public databases, was employed to validate the MS/MS fragments against the proposed chemical structure.

As shown in Table 1, the metabolites discriminating among groups were primarily polyunsaturated fatty acids (PUFAs). Notably, α-linolenic acid and related C18 lipid-class features were significantly downregulated in NMP-exposed plants. According to the literature, the predominant fatty acids in lettuce are linolenic acid and linoleic acid, comprising approximately 60% and 20% of the total fatty acids, respectively (Sularz et al., 2020) highlighting the potential metabolic impact of this alteration. Changes in fatty acid patterns have been observed in previous studies of plants under both biotic and abiotic stresses, such as drought, extreme temperatures, salinity, and pathogen attack (Zi et al., 2022). Evidence has shown that C18 unsaturated fatty acids (UFAs) play a key role in plant defense against various biotic and abiotic stressors, being involved both directly and indirectly in stress response mechanisms (He and Ding, 2020). Linolenic acid is a key component of chloroplast membranes in plants, particularly within galactolipids. It plays a central role in membrane integrity, lipid metabolism, photosynthesis, and stress signaling. Linolenic acid is synthesized through the fatty acid biosynthetic pathway, which is responsible for producing long-chain fatty acids. It is typically synthesized from oleic acid by a desaturation process catalyzed by ω-3 desaturases, producing α-linolenic acid (ALA), a key component of plant membranes. Under stress conditions, plants may degrade membrane lipids to release fatty acids for energy or signaling, which is consistent with the decreased linolenic acid levels observed in this study following NMP exposure. Additionally, reactive oxygen species (ROS) generated during stress have been reported to oxidize linolenic acid by lipoxygenases (LOX)-mediated pathways, although these parameters were not directly measured in the present study. Linolenic acid serves as a precursor for jasmonic acid (JA) and a range of bioactive compounds known as oxylipins (Savchenko et al., 2014), which are signaling molecules involved in responses to wounding, pathogen attack, and abiotic stress. These bioactive compounds also include 12-oxo-phytodienoic acid (OPDA), an intermediate in jasmonate biosynthesis that regulates stress responses, and hydroxy fatty acids and aldehydes, which are involved in signaling pathways related to stress and injury responses (Eckardt, 2008). In some stress scenarios, fatty acid synthesis may shift toward more stable saturated or monounsaturated forms, potentially reducing linolenic acid content. However, certain stress conditions may also trigger its accumulation as a protective mechanism. The outcome depends on the type and duration of stress, as well as the plant species. Overall, the consistent downregulation of α-linolenic acid and related C18 features observed here is consistent with their role in membrane integrity, lipid metabolism, and stress signaling in *Lactuca sativa*. Direct quantification of ROS, LOX activity, and jasmonate biosynthesis in future studies could provide additional support for the underlying mechanistic basis of these observations.

### 3.3 Influence of particle size (polystyrene NMPs) on the molecular mass distribution of PUFAs

**FIGURE 5:**
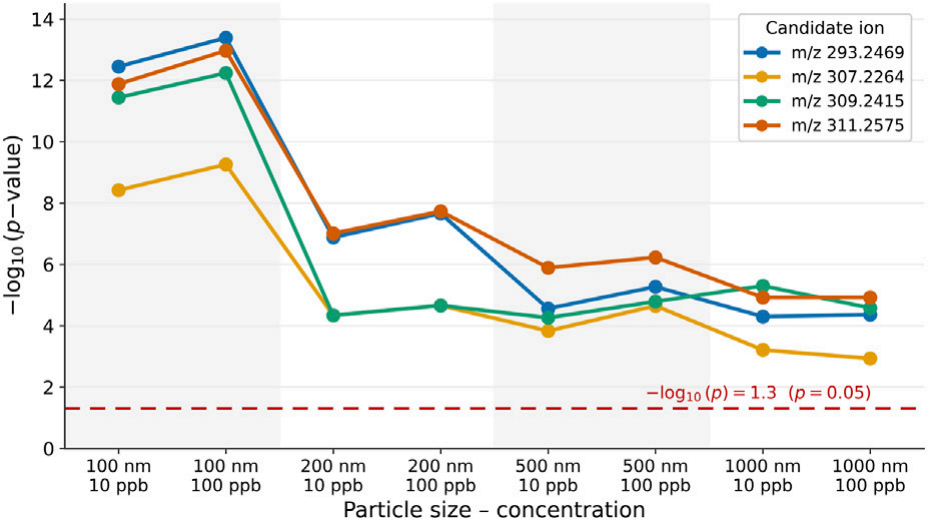
Statistical significance (-log p-value) by particle size (at 10 and 100 ppb) on the molecular mass distribution of PUFAs. Each point represents a Welch t-test comparing the unexposed control against plants exposed to the indicated size–concentration combination. Data provided in Supplementary Table S5.

The–log_10_ (p-value) graph shown in Figure 5 reveals statistically significant differences across all tested particle sizes, demonstrating that nanoparticle size plays a crucial role in the reduction of these metabolites. To assess the effect of nanoparticle size, statistical significance (-log p-value) was evaluated at two concentrations, 10 and 100 ppb. The lower concentrations were selected to enhance the visualization and interpretation of size-dependent effects while minimizing potential confounding from concentration-driven responses. The majority of data points were above the 1.3 threshold, confirming that changes in particle size had a measurable impact on chromatographic peak areas. However, the effect was not uniform across all particle sizes, indicating that certain sizes induced more pronounced changes than others.

Across the four candidate ions, statistical significance was greatest for the smallest particles and declined as particle size increased (full per-size values in Figure 5; Supplementary Table S5). The strongest responses occurred at 100 nm and 100 ppb, reaching -log10 p-values of 13.39 (293.2469) and 12.97 (311.2575); the 200 nm class was intermediate (-log10 p ≈ 6.9–7.7) and the 500 and 1000 nm classes were markedly weaker. The ions 311.2575 and 309.2415 retained moderate significance at the larger sizes (-log10 p ≈ 4.3–6.2), whereas 307.2264 and 293.2469 were the most size-sensitive (declining to -log10 p ≈ 2.9–4.3). Overall, particle size is a crucial determinant of metabolite reduction: the smallest particles, particularly 100 nm, drove the strongest downregulation across molecular masses, whereas the 500 and 1000 nm particles had much weaker effects.

### 3.4 Effect of polystyrene NMPs concentration on PUFAs

A separate analysis was conducted to evaluate the effect of concentration by comparing control samples with various concentration levels (10 ppb, 100 ppb, 300 ppb, and 50 ppm). The -log_10_ (p-value) values for each molecular mass were analyzed to assess the extent of concentration-induced changes (Table 2). This analysis helps identify which molecular masses are most sensitive to concentration variations and whether certain concentrations lead to more significant differences in chromatographic peak areas.

**TABLE 2:** Comparative analysis of molecular mass responses of PUFAs in control vs. concentration comparisons. -log10 (p-value) for control vs. concentration comparisons.

*m/z* (molecular mass)	-log10 (p-value) (control to 10 ppb)	-log10 (p-value) (control to 100 ppb)	-log10 (p-value) (control to 300 ppb)	-log10 (p-value) (control to 50 ppm)
307.2264	8.42	9.26	8.03	8.78
309.2415	11.44	12.24	8.51	11.69
293.2469	12.45	13.39	10.34	11.65
311.2575	11.88	12.97	10.70	11.83

The 100-ppb concentration exhibited the strongest statistical significance across all molecular masses, indicating that this concentration induces the most pronounced chromatographic changes. In contrast, the 300-ppb concentration consistently showed lower significance compared to 10 ppb and 100 ppb, suggesting a weaker effect. Higher concentrations (50 ppm) also had a strong impact, though their effect was slightly less pronounced than that of 100 ppb for most molecular masses. This nonlinear trend in concentration implies that an increase in concentration does not necessarily result in a stronger impact. As shown in Table 2, this pattern was consistent across all four molecular masses (293.2469, 307.2264, 309.2415 and 311.2575): the 100-ppb concentration produced the highest -log10 (p-value) in every case, the 300-ppb concentration the lowest, and the 10-ppb and 50-ppm concentrations intermediate values.

## 4 Conclusion

This study provides new insights into the metabolic disruption caused by nano- and microplastics (NMPs) in *Lactuca sativa*, with a particular focus on the impact of particle size on the molecular mass distribution of polyunsaturated fatty acids (PUFAs). Using a controlled hydroponic exposure system combined with advanced metabolomic techniques, we demonstrate that smaller nanoparticles (especially 100 nm) exert the most pronounced effects, leading to significant downregulation of PUFAs-related metabolites, most notably those associated with α-linolenic acid. Given that linolenic acid constitutes the major fraction of total fatty acids in lettuce and plays a central role in chloroplast membrane structure, lipid metabolism, photosynthesis, and stress signaling, its alteration may have serious implications for plant health and resilience. The observed metabolite shifts are consistent with known plant responses to abiotic stress, in which membrane lipids are degraded, and linolenic acid is oxidized or consumed in the biosynthesis of jasmonic acid and other oxylipins, which are key regulators of plant defense and adaptation.

Two features of this data highlight an important nuance. First, particle size, not concentration, governs the whole-plant response, echoing the size dependence of the metabolome, in which the smallest particles drive the strongest downregulation of PUFAs. However, the morphological and metabolomic profiles are not aligned: 100 nm particles perturbed the metabolome most while leaving height and root length intact, whereas 200 nm produced the clearest morphological suppression. This decoupling suggests that NMP-induced metabolic reprogramming (among it the depletion of membrane PUFAs and their stress-signalling derivatives) can precede any visible growth effect, so that a plant that looks healthy, or even larger, may already carry an altered biochemical profile. Second, because the most consistent change relative to the control was a gain in leaf number and biomass rather than a loss, morphology alone would understate the impact of NMP exposure; the metabolomic signature is both the more sensitive marker of stress and the more direct link to nutritional quality, since the affected PUFAs (α-linolenic acid foremost) are themselves primary determinants of the dietary value of leafy vegetables (Sularz et al., 2020).

These findings not only highlight the capacity of metabolomics to detect subtle biochemical changes triggered by environmental contaminants but also underscore the potential risk that nanoplastics pose to agricultural crops at the molecular level. The size-dependent effects observed here suggest that nanoparticle contamination, even at low concentrations, may interfere with essential lipid signaling and stress response pathways in plants. Future studies combining targeted lipidomics with direct measurement of reactive oxygen species, jasmonate quantification and fatty-acid-desaturase expression would be needed to confirm the proposed lipid-peroxidation/oxylipin involvement and to move from association to mechanism.

## Data Availability

The original contributions presented in the study are included in the article/Supplementary Material, further inquiries can be directed to the corresponding author.
