# Supplementary material for: Uncovering metabolomic dysregulation in Lactuca sativa caused by exposure to nano- and microplastics (NMPs)
Source: Front Toxicol. 2026 Jul 24;8:1872426. doi: 10.3389/ftox.2026.1872426 (PMC13447916; doi:10.3389/ftox.2026.1872426)
Supplement: Supplementary file 1 [file Supplementaryfile1.docx]

**Supplementary Information**

**Uncovering metabolomic dysregulation in *Lactuca sativa* caused by exposure to nano - and microplastics (NMPs)**

**María José Gómez-Ramos**^a^**, Harshit Sahai**^a^**, Chen Wang^b^, Hui Li^b^, Amadeo R. Fernández-Alba**^a^**, María Dolores Hernando Guil^c,*^**

^a^Pesticide Residues Research Group, Department of Chemistry and Physics, Research Centre for Mediterranean Intensive Agrosystems and Agri-Food, Biotechnology (CIAIMBITAL), University of Almeria, Agrifood Campus of International, Excellence, ceiA3, Almeria, E-04120, Spain

^b^ Institute of Environmental Pollution and Health, School of Environmental and Chemical Engineering, Shanghai University, 200444, Shanghai, China

^c^ Department of Desertification and Geo-ecology, Experimental Station of Arid Zones, CSIC, Ctra. Sacramento s/n, La Cañada de San Urbano, 04120, Almería, Spain.

*Corresponding author.

Corresponding author´s email address: [hernando.dolores@eeza.csic.es](mailto:hernando.dolores@eeza.csic.es)

# Supplementary Figures

**Figure S1.** Confirmation of α-linolenic acid identity by comparison with an authentic reference standard (1 ppm).

Confirmation of α-linolenic acid identity by comparison of LC-QTOF-MS/MS data with an authentic reference standard (1 ppm). Upper panel: extracted ion chromatogram (XIC), TOF-MS spectrum, and TOF-MS/MS IDA spectrum of the 1 ppm analytical standard, showing the [M+CH₃OH+H]⁺ adduct at m/z 311.2575 (molecular formula C₁₈H₃₀O₂, score = 86.2, mass error = 0.7 ppm) and a library Purity of Fit of 100%. Lower panel: corresponding data from a representative *Lactuca sativa* control sample at RT = 15.71 min (molecular formula C₁₈H₃₀O₂, score = 80.7, mass error = 1.5 ppm), with a library Purity of Fit of 98.2%, confirming structural identity with α-linolenic acid. Note: retention time comparison between standard and sample was not possible due to inter-batch chromatographic drift; identity confirmation is based on exact mass accuracy, isotope profile score, and MS/MS spectral Purity of Fit (98.2%).

Extracted ion chromatogram (XIC) overlays showing co-elution of the [M+H]⁺ precursor ion (m/z 279.232 ± 0.010 Da, pink) and the [M+CH₃OH+H]⁺ adduct ion (m/z 311.258 ± 0.010 Da, blue) at identical retention time in both the analytical standard (left) and the *Lactuca sativa* control sample (right), confirming that both ions originate from the same chromatographic peak.

**Figure S2.** Evidence for [M+CH₃OH+H]⁺ methanol adduct formation for the three Level 4 annotated compounds in *Lactuca sativa* samples. For each compound: extracted ion chromatogram (XIC) overlay showing co-elution of the [M+CH₃OH+H]⁺ adduct ion (blue) and the [M+H]⁺ precursor ion (pink) at identical retention time (±0.010 Da extraction window); SCIEX OS automatic [M+CH₃OH+H]⁺ ion assignment (inset, red box); TOF-MS spectrum confirming the adduct as the most abundant ion with the molecular formula assignment; and TOF-MS/MS IDA spectrum showing the characteristic neutral loss of 32.026 Da (CH₃OH, red arrow) confirming adduct identity.

**C₁₈H₂₈O₂ compound:** [M+CH₃OH+H]⁺ m/z 309.2415, [M+H]⁺ m/z 277.216 (32.8% R.A.), RT = 15.13 min, Mass error = −2.7 ppm.

**C₁₈H₂₆O₂ compound:** [M+CH₃OH+H]⁺ m/z 307.2264, [M+H]⁺ m/z 275.200 (29.7% R.A.), RT = 14.65 min, Mass error = −2.2 ppm

**C₁₈H₂₈O compound:** [M+CH₃OH+H]⁺ m/z 293.2469, [M+H]⁺ m/z 261.222 (8.0% R.A.), RT = 15.23 min, Mass error = 1.3 ppm.

**Supplementary Figure S3.** Negative / autofluorescence controls — representative CLSM images of unexposed (control) *Lactuca sativa* root (a) and leaf (b) tissues, acquired and processed identically to the NMP-exposed samples in Figures S4–S6 (Section 2.3): brightfield/grayscale (a1, b1), the PS-NP fluorescence channel as a maximum-intensity projection of the full z-stack (a2, b2), and the composite overlay (a3, b3). The fluorescence channel is displayed at a fixed low intensity range identical for both tissues; only weak, diffuse cell-wall autofluorescence is detectable — the residual background used to define the subtraction threshold applied to the exposed samples — with none of the discrete fluorescent puncta indicative of internalized particles, confirming the absence of PS-NP signal in control tissues. Scale bars, 200 µm.

**Supplementary Figure S4.** CLSM images of lettuce root samples highlighting detected PS-NPs accumulation. Brightfield or grayscale images of plant tissue (a1, b1 and c1), fluorescent channel in isolation containing only the PS-NP signal (a2, b2 and c2) and composite overlay of fluorescent and grayscale channels highlighting accumulation inside tissue (a3, b3 and c3). Images correspond to lettuce plant exposed to 50 mg/L of 100 nm particles (a and b) and 200 nm particles (c). Scale provided in the bottom right edge of each image.

**Supplementary Figure S5.** CLSM images of lettuce root samples highlighting detected PS-NPs accumulation. Brightfield or grayscale images of plant tissue (a1, b1), fluorescent channel in isolation containing only the PS-NP signal (a2, b2) and composite overlay of fluorescent and grayscale channels highlighting accumulation inside tissue (a3, b3). Images correspond to lettuce plant exposed to 50 mg/L of 500 nm particles (a) and 1000 nm particles (b). Scale provided in the bottom right edge of each image.

**Supplementary Figure S6.** CLSM images of lettuce leaf samples highlighting detected PS-NPs accumulation. Brightfield or grayscale images of plant tissue (a1, b1, c1, d1), fluorescent channel in isolation containing only the PS-NP signal (a2, b2, c2, d2) and composite overlay of fluorescent and grayscale channels highlighting accumulation inside tissue (a3, b3, c3, d3). Images correspond to lettuce plant exposed to 50 mg/L of 100 nm particles (a), 200 nm particles (b), 500 nm (c) and 300 µg/L of 1000 nm particles (d). Scale provided in the bottom right edge of each image.

**Figure S7. PCA**

**A)**

Principal Component Analysis (PCA) scores plot generated in MarkerView on the full normalised dataset.

Total area sum normalisation prior to feature filtering. Each point represents one sample: NMP-exposed plants (Case, dark green squares), control plants (Ctrl, light green squares), and pooled quality control samples (QC, blue circles). PC1 explains 45.4% and PC2 explains 18.1% of total variance. QC samples cluster tightly between the two biological groups, confirming satisfactory analytical reproducibility throughout the injection sequence. Clear separation of Control and Case groups along PC1 is observed independently of subsequent filtering steps.

**B)**

Unsupervised Principal Component Analysis (PCA) scores plot generated in MetaboAnalyst 6.0

Filtered dataset (1,630 features; blank subtraction ≥5×, isotope removal, and 80% prevalence filtering applied). Each point represents one sample: NMP-exposed plants (Case, pink) and control plants (Control, green). Ellipses represent 95% confidence intervals. PC1 explains 29.4% and PC2 explains 9.9% of total variance. Complete separation between Control and Case groups along PC1 is observed without any group label information, providing unsupervised evidence for NMP-induced metabolomic dysregulation in *Lactuca sativa*.

**Figure S8**

**A) PLS-DA**

Partial Least Squares Discriminant Analysis (PLS-DA) scores plot generated in MetaboAnalyst 6.0 on the filtered and normalised dataset (1,630 features; total area sum normalisation, blank subtraction ≥5×, isotope removal, and 80% prevalence filtering applied). Each point represents one sample: NMP-exposed plants (Case, pink), control plants (Control, green), and pooled quality control samples (QC, blue). Ellipses represent 95% confidence intervals. Component 1 explains 25.6% and Component 2 explains 5.4% of the variance. Complete separation between Control and Case groups is observed along Component 1. The tight clustering of QC samples confirms satisfactory analytical reproducibility throughout the injection sequence (mean CV% = 14.3% across retained features). Model validation metrics: R² = 0.998, Q² = 0.991 (leave-one-out cross-validation, 2 components), indicating robust class discrimination without overfitting (ΔR²−Q² < 0.01).

**B) PLS-DA cross validation**

PLS-DA cross-validation performance plot generated in MetaboAnalyst 6.0 on the filtered and normalized dataset: Accuracy, R², Q² for 1–5 components. R²=0.998, Q²=0.991 at 2 components.

# Supplementary Tables

**Table S1.** Confocal laser scanning microscopy (CLSM) acquisition and image-processing settings.

| **Parameter** | **Setting** |
| --- | --- |
| ***Image acquisition*** | |
| Microscope / software | Leica TCS SP8; LAS X |
| Objective | HC PL APO CS 10×/0.40 DRY |
| Zoom factor | 0.75 |
| Excitation / emission | 530 / 580 nm |
| Z-step size | 1 µm |
| Scan mode / speed | xyz; 400 Hz |
| Detector | HyD, standard mode; gain 117.1% |
| Time gating | 6.72–12 ns (autofluorescence reduction) |
| Background correction | Red-channel signal of unexposed control used to define and subtract the autofluorescence threshold, applied to exposed samples |
| ***Instrument QA/QC*** | |
| Reference beads | TetraSpeck Microspheres (ThermoFisher T7282), 1.0 µm |
| Laser-power verification | Optical power meter (PM 160, Thorlabs) |
| Quality standard | UNE-EN ISO 9001 |
| ***Image processing (Fiji-ImageJ)*** | |
| Projection | Z-stack maximum-intensity |
| Channels | Colour channels separated for individual analysis |
| Background subtraction | Subtract function applied until no control fluorescence remained; threshold then applied to exposed samples |
| Contrast enhancement | Orange-hot lookup table |

**Table S2.** LC–QTOF-MS/MS operating parameters.

| **Parameter** | **Setting** |
| --- | --- |
| ***Liquid chromatography*** | |
| Analytical column | Agilent Zorbax Eclipse Plus C8, 100 × 2.1 mm, 1.8 µm |
| Guard column | Zorbax Eclipse Plus C18, 2.1 × 5 mm, 1.8 µm |
| Flow rate | 300 µL/min |
| Column temperature | 35 °C |
| Injection volume | 5 µL |
| Mobile phase A | Water/methanol 98:2 (v/v) |
| Mobile phase B | Methanol/water 98:2 (v/v) |
| Additives (A and B) | 5 mM ammonium formate; 0.1% (v/v) formic acid |
| Gradient (%A) | 100% (0–2 min) → 70% (3 min) → 50% (6 min) → 0% (16–19 min) → 100% (19–23 min, re-equilibration) |
| Total run time | 23 min |
| ***Mass spectrometry — X500R QTOF, positive ESI*** | |
| Source / probe | Turbo V; TwinSprayer |
| Ion source gas 1 | 40 psi |
| Ion source gas 2 | 50 psi |
| Curtain gas | 25 psi |
| CAD gas | 7 |
| Source temperature | 450 °C |
| Spray voltage | 5500 V |
| TOF resolution | 32,000 fwhm at m/z 200 |
| Acquisition mode | IDA: TOF-MS survey m/z 80–950; dependent MS/MS m/z 50–950 |
| IDA criteria | ≤10 candidate ions; intensity threshold 200 cps; dynamic background subtraction on; dynamic exclusion 6 s after one occurrence |
| Declustering potential | 80 V |
| Collision energy | 35 ± 15 V |
| Accumulation time | 0.1 s |
| Mass calibration | Automatic external, every 5 samples (mass accuracy < 2 ppm) |

**Table S3.** Untargeted data-processing parameters (SCIEX Explorer/Analytics and MarkerView).

| **Parameter** | **Setting** |
| --- | --- |
| ***Acquisition / processing software*** | |
| Software | Explorer and Analytics (v3.0.0.3, AB Sciex) |
| ***Non-target screening — peak detection*** | |
| Noise threshold | 700 counts |
| Precursor mass accuracy | ± 5 ppm |
| Signal-to-noise (S/N) | ≥ 3 |
| Minimum peak height | 700 counts |
| Fragment-ion mass error | ± 15 ppm |
| ***Formula assignment (formula finder)*** | |
| Elements considered | C, H, N, O, S |
| Mass tolerance | 10 ppm |
| Area ratio (sample/control) | 10 |
| Additional criteria | Isotope-pattern distribution; ring-and-double-bond equivalents; MS/MS interpretation |
| ***MarkerView feature finding (v1.3.1, SCIEX)*** | |
| Subtraction offset | 10 scans |
| Subtraction multiplication factor | 1.2 |
| Minimum RT peak width | 3 scans |
| Minimum spectral peak width | 5 ppm |
| Noise threshold | 70 |
| ***Alignment*** | |
| Retention-time tolerance | 0.5 min |
| Mass tolerance | 10 ppm |
| Intensity threshold | 70 |
| Maximum peaks | 5000 |

**Table S4.** Morphological traits of Lactuca sativa after 20-day hydroponic exposure to polystyrene NMPs (mean ± SD).

| **Treatment** | **n** | **Plant height (cm)** | **Plant fresh weight (g)** | **Root length (cm)** | **Number of leaves** |
| --- | --- | --- | --- | --- | --- |
| **Control** | 5 | **18.4 ± 1.9** | **3.81 ± 0.60** | **8.6 ± 1.8** | **7.0 ± 1.0** |
| 100 nm, 10 µg/L | 5 | 19.2 ± 1.9 | 4.66 ± 0.13 | 9.1 ± 1.5 | 10.6 ± 0.5^***^ |
| 100 nm, 100 µg/L | 5 | 20.0 ± 0.9 | 5.06 ± 0.72 | 10.0 ± 1.8 | 9.4 ± 1.1 |
| 100 nm, 300 µg/L | 5 | 18.0 ± 1.0 | 4.57 ± 0.74 | 8.6 ± 0.9 | 10.2 ± 0.8^**^ |
| 100 nm, 50 mg/L | 4 | 19.1 ± 1.3 | 4.71 ± 0.51 | 9.1 ± 1.4 | 9.8 ± 0.5^*^ |
| 200 nm, 10 µg/L | 6 | 16.4 ± 1.8 | 4.89 ± 0.84 | 7.1 ± 1.0 | 10.2 ± 1.8^***^ |
| 200 nm, 100 µg/L | 7 | 17.0 ± 2.7 | 5.05 ± 0.94 | 8.2 ± 1.6 | 10.6 ± 1.0^****^ |
| 200 nm, 300 µg/L | 7 | 15.4 ± 2.2 | 3.90 ± 0.68 | 7.4 ± 2.3 | 9.1 ± 1.2 |
| 200 nm, 50 mg/L | 5 | 14.4 ± 1.5 | 4.11 ± 0.88 | 6.1 ± 0.7 | 9.2 ± 0.8 |
| 500 nm, 10 µg/L | 5 | 18.0 ± 2.6 | 4.20 ± 0.47 | 7.7 ± 1.6 | 9.0 ± 0.7 |
| 500 nm, 100 µg/L | 5 | 19.9 ± 1.1 | 4.01 ± 0.99 | 9.6 ± 0.9 | 8.4 ± 2.1 |
| 500 nm, 300 µg/L | 5 | 19.4 ± 0.8 | 4.05 ± 0.59 | 9.5 ± 0.8 | 7.6 ± 0.5 |
| 500 nm, 50 mg/L | 5 | 19.6 ± 1.6 | 4.41 ± 0.39 | 9.8 ± 1.7 | 9.6 ± 0.5^*^ |
| 1000 nm, 10 µg/L | 5 | 20.4 ± 1.6 | 5.66 ± 0.38^**^ | 9.8 ± 1.3 | 11.0 ± 0.7^****^ |
| 1000 nm, 100 µg/L | 5 | 20.1 ± 2.4 | 5.07 ± 0.89 | 9.7 ± 1.1 | 10.4 ± 1.3^***^ |
| 1000 nm, 300 µg/L | 5 | 19.6 ± 1.0 | 4.69 ± 0.66 | 10.0 ± 0.9 | 9.2 ± 0.8 |
| 1000 nm, 50 mg/L | 5 | 17.6 ± 2.4 | 3.98 ± 0.41 | 8.4 ± 2.3 | 9.2 ± 0.4 |

Values are mean ± SD of n = 4–7 individual plants per group (n given). The two-way ANOVA used Type III sums of squares (appropriate for the unequal group sizes).

*, **, ***, **** indicate a significant difference from the unexposed control by Tukey’s HSD following one-way ANOVA across all 17 groups (p < 0.05, < 0.01, < 0.001, < 0.0001, respectively). No treatment differed significantly from the control for plant height or root length.

One-way ANOVA (17 groups): height F(16,72) = 4.79, p < 0.0001, R² = 0.52; fresh weight F(16,72) = 3.05, p = 0.0006, R² = 0.40; root length F(16,72) = 3.08, p = 0.0006, R² = 0.41; leaf number F(16,72) = 5.10, p < 0.0001, R² = 0.53.

Two-way ANOVA (16 size × concentration treatments): particle diameter was the dominant factor for every trait — height 41 % of variance (p < 0.0001), root length 29 % (p < 0.0001), leaf number 17 % (p = 0.0004), fresh weight 10 % (p = 0.013). Concentration was significant only for fresh weight (11 %, p = 0.012) and leaf number (11 %, p = 0.008); the size × concentration interaction was significant only for leaf number (p = 0.043).

**Table S5.** Influence of nanoparticle size on metabolite reduction. Statistical significance (-log p-value) by size (concentration at 10 and 100 ppb)

| **Exposure NPs**  **(size, nm)** | **Level**  **(ng/g)** | **293.2469**  **-log10(p-value)** | **311.2575**  **-log10(p-value)** | **307.2264**  **-log10(p-value)** | **309.2415**  **-log10(p-value)** |
| --- | --- | --- | --- | --- | --- |
| 100 | 10 | 12.45 | 11.88 | 8.42 | 11.44 |
|  | 100 | 13.39 | 12.97 | 9.26 | 12.24 |
| 200 | 10 | 6.88 | 7.01 | 4.34 | 4.34 |
|  | 100 | 7.66 | 7.73 | 4.66 | 4.66 |
| 500 | 10 | 4.56 | 5.89 | 3.83 | 4.26 |
|  | 100 | 5.27 | 6.23 | 4.65 | 4.79 |
| 1000 | 10 | 4.30 | 4.92 | 3.21 | 5.30 |
|  | 100 | 4.36 | 4.92 | 2.93 | 4.58 |

# Supplementary Text S1

**Pre-processing and quality control**

Prior to multivariate analysis, all peak areas were normalized by total area sum in MarkerView. The normalized feature list was subsequently filtered by: (i) removal of isotopic signals automatically identified by MarkerView; (ii) blank subtraction, retaining only features with mean peak areas at least 5-fold higher than procedural blank samples; and (iii) prevalence filtering, retaining features detected in at least 80% of samples within at least one experimental group. Analytical reproducibility was assessed using pooled QC samples (n = 8) injected at regular intervals throughout the analytical sequence. CV% was calculated for each feature as the ratio of standard deviation to mean peak area across QC injections.

**Statistical analysis**

Multivariate and univariate analyses were performed by comparing all control samples against all NMP-exposed samples pooled across particle sizes and concentrations, in order to identify metabolic features discriminant between exposed and unexposed plants. The concentration- and size-dependent behavior of the identified discriminant features was subsequently characterized within the NMP-exposed group. Initial exploratory analysis was conducted in MarkerView™ software (v1.3.1, SCIEX). MarkerView™ provided graphical tools for rapid data review, including volcano plots of p-value versus log fold change to identify features with low p-values and large fold changes, and profile plots to visualize the intensity of selected compounds across samples. Subsequent statistical validation and multivariate analysis were performed using MetaboAnalyst 6.0 (https://www.metaboanalyst.ca). PLS-DA was performed on the filtered dataset (1,630 features) with the number of components optimized based on leave-one-out cross-validation (LOO-CV) performance. Model performance was evaluated by R² (goodness of fit) and Q² (predictive ability) obtained by LOO-CV. Statistical significance of individual features was assessed by two-sample Welch t-test with Benjamini-Hochberg FDR correction applied to the full feature list (significance threshold: q < 0.05).
